# Defining left bundle branch block according to the new 2021 European Society of Cardiology criteria

**DOI:** 10.1007/s12471-022-01697-5

**Published:** 2022-05-03

**Authors:** S. Calle, F. Timmermans, J. De Pooter

**Affiliations:** Department of Cardiology, 8-K12, University Hospital Ghent, Ghent, Belgium

**Keywords:** Left bundle branch block, Cardiac resynchronisation therapy

## Abstract

Correctly diagnosing left bundle branch block (LBBB) is fundamental, as LBBB occurs frequently in heart failure and may trigger a vicious cycle of progressive left ventricular dysfunction. Moreover, a correct diagnosis of LBBB is pivotal to guide cardiac resynchronisation therapy. Since the LBBB diagnostic criteria were recently updated by the European Society of Cardiology (ESC), we assessed their diagnostic accuracy compared with the previous ESC 2013 definition. We further discuss the complexity of defining LBBB within the context of recent insights into the electromechanical pathophysiology of LBBB.

Left bundle branch block (LBBB) was first recorded electrocardiographically in humans in 1914 [1]. Multiple criteria to define LBBB have been proposed, based on experimental canine studies, human case studies, intracardiac mapping, observations in cardiac resynchronisation therapy (CRT) responders and characteristics of transcatheter aortic valve replacement (TAVR)-induced LBBB [2]. Although the main features of contemporary LBBB definitions are similar (i.e. QRS prolongation, dominant S waves in lead V1 and lateral notching or slurring), differences in definitions were shown to result in significant discordance when scoring LBBB in clinical practice [2–6]. This is a remarkable observation, given that most LBBB definitions are derived from the same 1985 World Health Organisation consensus criteria [2, 7]. However, correct electrocardiographic assessment of LBBB is fundamental, as ‘true’ LBBB is associated with the presence of LBBB-induced dyssynchrony [3] and improves selection of patients eligible for CRT [4, 6, 8].

Current controversy in defining LBBB is primarily related to the difficulties in identifying patients with a typical LBBB activation that is characterised by a reversed, right-to-left septal depolarisation [2]. Because studies over the past century have included patients with various types of conduction delay (proximal vs distal, focal vs diffuse), this probably resulted in heterogeneous electrocardiographic LBBB criteria. More recent studies consistently showed that the European Society of Cardiology (ESC) 2013 [9] and the Strauss [1] definitions had the highest sensitivity for predicting both echocardiographic and clinical response to CRT, whereas the American Heart Association (AHA) definition [10] had the highest specificity [4, 6], suggesting that the highly selective AHA definition may be too stringent. In patients with TAVR-induced LBBB, similar findings were observed, with the ESC 2013 and Strauss definitions providing a higher sensitivity to identifying LBBB than the AHA definition [2].

Recently, the ESC proposed new electrocardiographic criteria to define LBBB [11]. The updated ESC 2021 definition emphasises on the importance of QRS notching/slurring and delayed R‑wave peak time, and provides new recommendations on ST-segment and T‑wave assessment. However, how the 2021 revised definition performs in diagnosing LBBB compared with the previous ESC 2013 definition has not been addressed.

We compared the diagnostic criteria of the ESC 2013 and ESC 2021 definitions in a general LBBB population. Consecutive patients with LBBB and varying left ventricular ejection fraction (LVEF) underwent a prospective electro- and echocardiographic examination at Ghent University Hospital from October 2018 through September 2021. LBBB was defined according to conventional criteria (QRS duration ≥ 120 ms, QS or rS in lead V1 and absence of Q waves in leads V5 and V6). Electrocardiograms were digitally stored in MUSE (GE Healthcare, USA) and continuous electrocardiographic characteristics were digitally analysed by the Marquette 12SL algorithm (GE Healthcare, USA). Septal flash on echocardiography was required to substantiate the presence of a true electromechanical LBBB substrate [12, 13]. CRT was implanted according to contemporary ESC guidelines [9, 11]. The study was approved by the Ethics Committee of Ghent University Hospital.

The LBBB cohort consisted of 281 patients (mean age 68 ± 13 years, 56% male, coronary artery disease 25%). Mean LVEF was 47 ± 14%, with 21% of patients having an LVEF ≤ 35%. Whereas 100% of patients met all the ESC 2013 criteria in this cohort, only 12% of patients met the complete set of ESC 2021 criteria (Tab. 1, Fig. 1). From this cohort, 61 patients underwent CRT implantation, of which 27 (44%) patients were categorised as CRT super-responders, based on improvement in LVEF from ≤ 35% to > 45% after ≥ 6 months of CRT. By definition, CRT super-responders represent unequivocal LBBB patients, as they display LBBB and septal flash, and completely reverse remodel, featuring a strong deterministic relationship between LBBB and reversible left ventricular remodelling in these patients. However, even among CRT super-responders, concordance with the ESC 2021 definition remained as low as 19% (Tab. 1).

**Table 1:** Comparison of European Society of Cardiology 2013 and 2021 criteria for left bundle branch block

Criteria		LBBB cohort (*n* = 281)	CRT super-responders (*n* = 27)
*ESC 2013 definition*		*281 (100)*	*27 (100)*
1	QRS duration ≥ 120 ms	281 (100)	27 (100)
2	QS or rS in lead V1	281 (100)	27 (100)
3	Broad (frequently notched or slurred) R waves in leads I, aVL, V5, or V6	281 (100)	27 (100)
4	Absent Q waves in leads V5 and V6	281 (100)	27 (100)
*ESC 2021 definition*		* 33 (12)*	* 5 (19)*
1	QRS ≥ 120 ms	281 (100)	27 (100)
2	Notches or slurring in the middle third of QRS in at least two of the following leads: V1, V2, V5, V6, I, and aVL—with a prolongation at the delayed peak in R in V5–V6 to longer than 60 ms	88 (31)	11 (41)
3	Generally, the ST segment is slightly opposed to the QRS polarity, and particularly when it is at least 140 ms and is rapidly followed by an asymmetrical T wave also of opposed polarity	281 (100)	27 (100)
4	Horizontal plane: QS or rS in V1 with small ‘r’ with ST slightly elevated and positive asymmetrical T wave and unique R wave in V6 with negative asymmetric T wave. When the QRS is less than 140 ms, the T wave in V6 may be positive	89 (32)	8 (30)
5	Frontal plane: exclusive R wave in I and aVL often with a negative asymmetrical T wave, slight ST depression, and usually QS in aVR with positive T wave	214 (76)	19 (70)
6	The QRS axis is variable	281 (100)	27 (100)

Values are *n* (%)

*CRT* cardiac resynchronisation therapy, *ESC* European Society of Cardiology, *LBBB* left bundle branch block

**Fig. 1:**
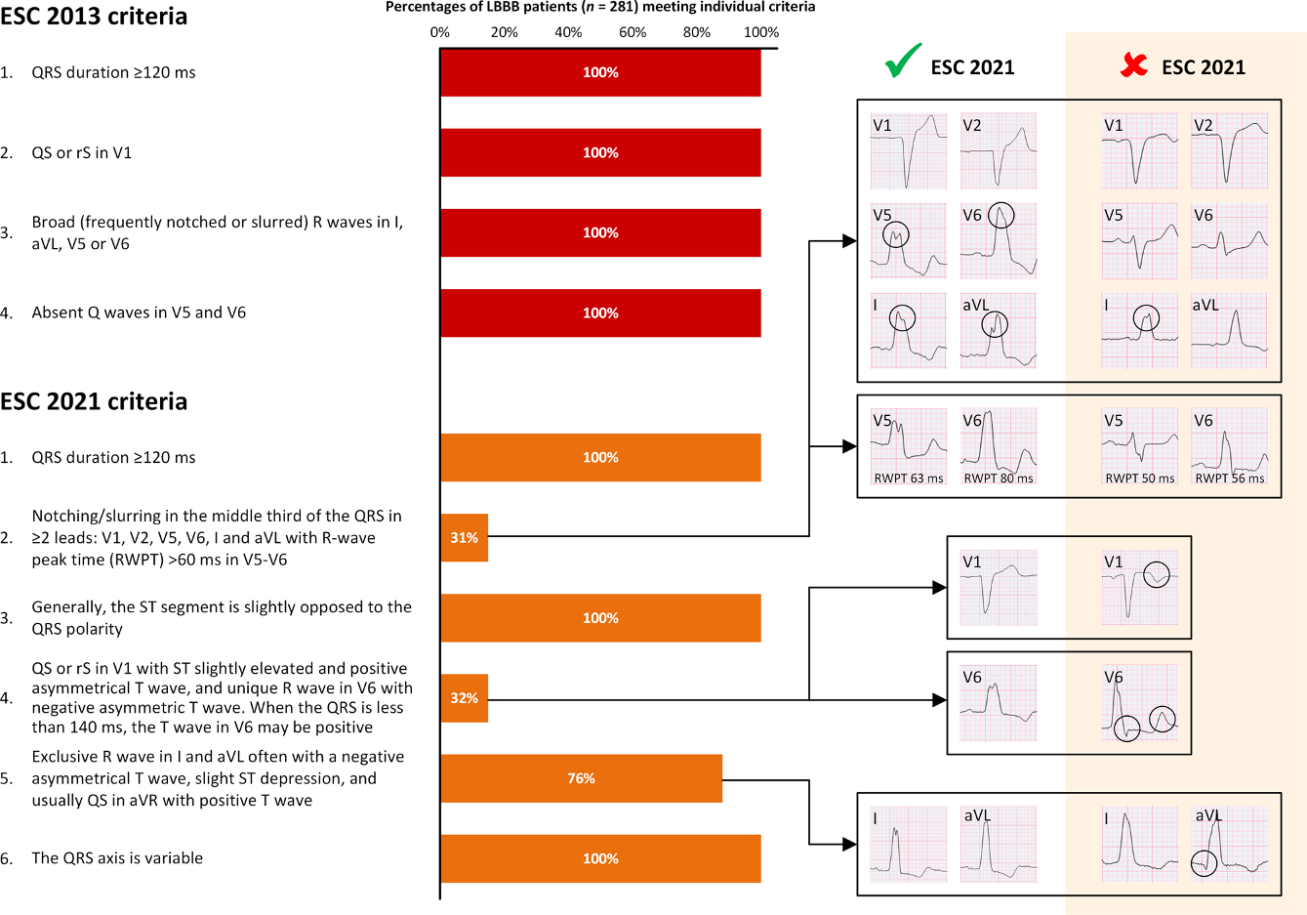
Defining left bundle branch block according to the European Society of Cardiology 2013 and 2021 criteria

The ESC 2013 definition identifies significantly more LBBB patients than the ESC 2021 definition. This probably relates to the extensive and more stringent criteria proposed in the new ESC 2021 LBBB definition. Previous studies favour the incorporation of QRS notching/slurring, which was consistently shown to be a hallmark for electrocardiographic LBBB [2–4, 6, 8, 14]. However, the use of too selective criteria for diagnosing LBBB might cause underdiagnosis of LBBB, as shown by our analysis. From a pathophysiological (i.e. identification of patients with right-to-left septal activation) and clinical (i.e. CRT eligibility and prediction of CRT response) point of view, broad LBBB inclusion criteria, including lateral QRS notching/slurring, seem reasonable to achieve high sensitivity. In addition, ancillary electro- and echocardiographic criteria, such as a delayed R‑wave peak time, a leftward oriented QRS axis and the presence of septal flash, apical rocking [15] or specific septal strain patterns [16] may be considered to improve the specificity and achieve maximal accuracy for diagnosing LBBB [17]. This two-tiered multi-modality approach in defining LBBB reflects the evolving insights into LBBB pathophysiology, causing a shift from a pure electrocardiographic definition towards LBBB as a clinical entity.
